# Anti-PF4/heparin antibodies associated with repeated hemofiltration-filter clotting: a retrospective study

**DOI:** 10.1186/cc6937

**Published:** 2008-06-25

**Authors:** Sigismond Lasocki, Pascale Piednoir, Nadine Ajzenberg, Arnaud Geffroy, Abdel Benbara, Philippe Montravers

**Affiliations:** 1 Département d'Anesthésie – Réanimation Chirurgicale, APHP, CHU Bichat-Claude Bernard, Université Paris 7, Paris, France; 2 Département d'Hématologie, APHP, CHU Bichat-Claude Bernard, Université Paris 7, Paris, France

## Abstract

**Introduction:**

Heparin-induced thrombocytopenia is an immune-mediated adverse drug reaction that is associated with a procoagulant state and both arterial and venous thrombosis. After observing two cases of repeated hemofiltration-filter clotting associated with high anti-PF4/heparin antibody concentrations, we systematically measured the anti-PF4/heparin antibody concentration in all cases of unexpected and repeated hemofiltration-filter clotting during continuous veno-venous hemofiltration (CVVH). The aim of this study was to identify factors associated with positive anti-PF4/heparin antibody in the case of repeated hemofiltration-filter clotting.

**Methods:**

We reviewed the charts of all patients who had an anti-PF4/heparin antibody assay performed for repeated hemofiltration-filter clotting between November 2004 and May 2006 in our surgical intensive care unit. We used an enzyme-linked immunoabsorbent assay (heparin-platelet factor 4-induced antibody) with an optical density (OD) of greater than 1 IU considered positive.

**Results:**

During the study period, anti-PF4/heparin antibody assay was performed in 28 out of 87 patients receiving CVVH. Seven patients were positive for anti-PF4/heparin antibodies (OD 2.00 [1.36 to 2.22] IU) and 21 were antibody-negative (OD 0.20 [0.10 to 0.32] IU). Baseline characteristics, platelet counts, and activated partial thromboplastin time ratios were not different between the two groups. CVVH duration was significantly decreased in antibody-positive patients (5.0 [2.5 to 7.5] versus 12.0 [7.5 to 24.0] hours; *P *= 0.007) as was CVVH efficiency (urea reduction ratio 17% [10% to 37%] versus 44% [30% to 52%]; *P *= 0.04) on heparin infusion. Anti-PF4/heparin antibody concentration was inversely correlated with CVVH duration. The receiver operating characteristic curve showed that a 6-hour cutoff was the best CVVH session duration to predict a positive antibody test (sensitivity 71%, specificity 85%, and area under the curve 0.83). CVVH duration (32 [22 to 37] hours; *P *< 0.05) and urea reduction (55% [36% to 68%]; *P *< 0.03) were restored by danaparoid sodium infusion.

**Conclusion:**

Repeated hemofiltration-filter clotting in less than 6 hours was often associated with the presence of anti-PF4/heparin antibodies, regardless of the platelet count. In antibody-positive patients, replacement of heparin by danaparoid sodium allowed the restoration of CVVH duration and efficiency.

## Introduction

Heparin-induced thrombocytopenia (HIT) is an antibody-mediated adverse effect of heparin. The initial description concerned arterial thrombosis during unfractionated heparin (UFH) therapy [1]. HIT subsequently has been associated with an increased risk of venous or arterial thrombosis [2-4]. The pathophysiology of HIT consists of the generation of anti-PF4/heparin antibodies, resulting in platelet and endothelial cell activation, leading to a procoagulant state [5]. Continuous veno-venous hemofiltration (CVVH) is widely used for renal replacement. Because UFH often is used as anticoagulation therapy, patients undergoing CVVH might be at risk for anti-PF4/heparin antibody generation or HIT. One clinical feature of HIT in this context could be repeated hemofiltration-filter clotting [6,7]. In our surgical intensive care unit (ICU), we recently observed two cases of HIT responsible for repeated hemofiltration-filter clotting in the absence of typical thrombocytopenia [8] and we then systematically measured the plasma anti-PF4/heparin antibody concentration in all patients with repeated hemofiltration-filter clotting during CVVH, with no obvious cause. The purpose of this study was to report a series of patients in whom an anti-PF4/heparin antibody test was performed and to identify factors associated with a positive test in this particular setting in order to determine when one should perform the test.

## Materials and methods

### Patients

Between 1 November 2004 and 1 May 2006, 87 patients underwent CVVH. Twenty-eight of these patients experienced repeated (≥ 2) hemofiltration-filter clotting before the scheduled end of the CVVH session (24 to 48 hours) with no obvious cause and anti-PF4/heparin antibodies were assayed. We reviewed the charts of these 28 patients. In accordance with French law, because of the retrospective nature of the study, no written consent was requested. The study was approved by our institutional review board. For each patient, general characteristics, platelet counts at various time points (admission, nadir [lowest reported concentration], day of anti-PF4/heparin antibody assay, and maximal count), and CVVH session duration and efficiency (assessed by the urea reduction ratio before/after each session) were recorded. The duration of CVVH sessions was obtained from the CVVH surveillance sheet, on which nurses routinely recorded data on an hourly basis. For each patient, an objective clinical probability scoring system was used to calculate the likelihood of HIT according to the Four T-score [9]. For this purpose, we attributed 1 point for the third item, 'Thrombosis', considering that the hemofiltration-filter clotting was equivalent to a 'recurrent thrombosis'.

### Continuous veno-venous hemofiltration settings

CVVH was performed using the Aquarius system (Aquarius; Edwards Lifesciences, Maurepas, France). Blood flow was set at 250 to 300 mL/minute, ultrafiltrate flow at 35 mL/kg per hour, and substitution solutions (Hemosol^® ^or Prismasol^®^; Hospal, Lyon, France) were delivered 1/3 pre-filter and 2/3 post-filter. A dual-lumen 11.5-French catheter (Mahurkar™; Tyco Healthcare, Wollerau, Switzerland) was inserted into either the femoral or right internal jugular vein. To exclude catheter dysfunction, catheters were changed in all patients at least once before anti-PF4/heparin antibody assay.

### Anticoagulation management

Before starting the CVVH session, the hemofiltration circuit was heparinized by priming with 10,000 IU of UFH (Héparine Choay^®^; Sanofi-Synthélabo, Paris, France) in 2,000 mL of saline solution. UFH then was continuously infused in the hemofiltration circuit to obtain an activated partial thromboplastin time (aPTT) ratio ranging between 1.2 and 2. The aPTT ratios were measured systematically from the arterial line. In the case of a positive PF4 test, UFH was replaced by danaparoid sodium (Orgaran^®^; Organon SA, Eragny-sur-Epte, France) using the manufacturer's recommendations to obtain a specific danaparoid anti-Xa activity of between 0.4 and 0.6 IU/mL.

### PF4 test and heparin-induced platelet activation assay

Anti-PF4/heparin antibodies were measured by enzyme-linked immunoabsorbent assay (heparin-platelet factor 4-induced antibody, HPIA; Diagnostica Stago, Asnières, France) according to the manufacturer's recommendations. The anti-PF4/heparin antibody was considered positive (PF4^+^) when optical densities (ODs) were greater than 1 IU. For anti-PF4/heparin antibody-positive patients, a platelet-rich plasma (PRP) aggregation assay was performed. Briefly, a plasma sample was incubated with donor PRP and a therapeutic (0.5 to 1 IU/mL) or high (100 IU/mL) UFH concentration in an aggregometer (Chronolog 490; Kordia Life Sciences, Leiden, The Netherlands) at 37°C. Platelet aggregation was monitored for 20 minutes. Results were considered positive if the aggregation was greater than or equal to 20% with a therapeutic UFH concentration and absent with a high UFH concentration. Appropriate positive and negative controls (stored patient plasma) were run in parallel with plasma samples. Cross-reactivity with danaparoid sodium was recorded under the same conditions.

### Statistical analysis

Data are presented as median (interquartile range) and compared with a Mann-Whitney or a chi-square test. Patients were classified into two groups (PF4^+ ^and PF4^-^) according to anti-PF4/heparin antibody concentration. For PF4^+ ^patients, duration and efficiency of CVVH sessions under UFH and danaparoid were compared with a Wilcoxon rank test. A receiver operating characteristic (ROC) curve was used to determine the best cutoff value for CVVH duration to predict a PF4^+ ^test. All statistics were performed with the MedCalc^® ^software (MedCalc Software, Mariakerke, Belgium). A *P *value of less than 0.05 was considered significant.

## Results

### Patients' characteristics

Among the 28 patients with unexplained hemofiltration-filter clotting, seven were anti-PF4/heparin antibody-positive. Baseline characteristics were not different between the two groups (PF4^+ ^or PF4^-^), except for a higher SAPS II (Simplified Acute Physiologic Score II) for PF4^- ^patients (Table 1). None of the patients had a Four T-score of less than 4, indicating that they all had an intermediate or high pre-test probability of HIT. A Four T-score of at least 7 had a positive predictive value of 100% and a negative one of 84% for the diagnosis of HIT, with a sensitivity of 43%. Four of the seven PF4^+ ^patients (Table 1, #1–4) also had a positive PRP aggregation assay and may be considered as having a very likely diagnosis of HIT [2,6]. Among the seven patients with HIT, two also had a vascular thrombosis, leading to the diagnosis of HITTS (heparin-induced thrombocytopenia and thrombosis syndrome): one had an ischemic stroke (#4) and the other one a pulmonary embolism (#5).

**Table 1:** Characteristics of the study population

										Time to onset, days				Platelet counts, 10^9^/L			aPTT ratios				
																	
	Patient	Gender	Age, years	SAPS II	SOFA score	Type of surgery	Sepsis	Shock	MV	ICU	CVVH	UFH	ICU length of stay, days	D_0_	D_PF4_	Four T-score	Mean	≥ 1.2, %	CVVH duration, hours	PF4, OD	Death
PF4^+^	1	M	54	34	12	Cardiac	No	Yes	Yes	8	6	8	22	42	10	5	1.2	50	14	1.18	No
	2	M	59	29	6	Cardiac	No	No	No	6	6	9	13	76	51	7	1.2	100	2	2.26	No
	3	M	63	50	13	Cardiac	Yes	Yes	Yes	9	8	9	28	101	20	7	1.3	33	4	2.68	No
	4	F	49	44	11	Cardiac	No	Yes	Yes	7	7	8	8	251	95	7	1.2	33	6	1.89	Yes
	5	M	62	86	16	Trauma	Yes	Yes	Yes	15	13	14	22	163	66	5	2.4	100	5	1.18	Yes
	6	M	55	52	12	Cardiac	No	Yes	Yes	10	8	11	26	144	200	4	1.9	100	2	2.00	No
	7	M	57	42	14	Cardiac	Yes	Yes	Yes	16	15	20	43	78	46	5	2.6	100	8	2.11	No

Total (n = 7)		6 M (86%)	57 (55–62)	44^a ^(36–51)	12.5 (12–14)	6 cardiac (86%)	3 (43%)	6 (86%)	6 (86%)	9 (8–15)	8 (6–13)	9 (9–14)	22 (18–28)	101 (77–158)	51 (26–88)	5 (5–7)	1.3 (1.2–2.4)	100 (50–100)	5^a ^(2.5–7.5)	2.00^a ^(1.36–2.22)	2 (40%)

PF4^-^	8	F	78	64	11	Visceral	Yes	Yes	Yes	4	2	5	4	109	48	6	3.1	100	19	0.11	Yes
	9	M	51	51	8	Visceral	Yes	Yes	Yes	5	5	5	16	413	234	6	1.6	75	4	0.19	No
	10	M	58	65	19	Cardiac	Yes	Yes	Yes	3	2	10	26	214	42	6	1.2	67	10	0.40	No
	11	M	77	68	15	Cardiac	Yes	Yes	Yes	14	14	24	47	176	52	5	1.8	100	12	0.13	Yes
	12	M	78	56	16	Cardiac	No	Yes	Yes	4	4	5	15	100	22	5	1.7	100	12	0.26	Yes
	13	M	83	89	18	Cardiac	Yes	Yes	Yes	4	3	5	28	86	33	6	1.8	100	9	0.17	Yes
	14	M	78	59	11	Cardiac	Yes	Yes	Yes	5	2	4	14	141	31	6	1.8	100	10	0.07	Yes
	15	M	37	35	14	Trauma	Yes	Yes	Yes	9	7	7	25	146	63	4	1.1	25	7	0.09	No
	16	F	73	62	15	Cardiac	Yes	Yes	Yes	36	36	39	50	94	48	5	3.9	100	24	0.29	Yes
	17	M	79	59	10	Visceral	Yes	Yes	Yes	9	9	9	12	169	86	6	1.4	100	31	0.18	Yes
	18	M	60	49	10	Cardiac	No	No	Yes	5	3	5	14	60	39	5	1.3	100	24	0.09	No
	19	M	48	48	8	Visceral	Yes	Yes	Yes	6	2	9	27	258	167	6	1.8	100	24	0.08	Yes
	20	F	75	54	11	Visceral	Yes	Yes	Yes	6	4	7	12	86	21	6	1.9	100	4	0.34	Yes
	21	M	60	39	13	Cardiac	No	Yes	Yes	4	3	5	58	61	47	6	1.5	100	24	0.19	No
	22	M	75	56	13	Cardiac	Yes	Yes	Yes	19	17	20	37	105	45	5	1.2	25	8	0.83	Yes
	23	M	78	57	13	Cardiac	Yes	Yes	Yes	5	4	6	61	67	44	6	1.0	0	13	0.02	No
	24	M	64	73	14	Visceral	No	Yes	Yes	16	16	16	26	206	95	6	1.4	100	16	0.24	No
	25	M	38	71	20	Vascular	Yes	Yes	Yes	4	4	4	33	210	49	6	1.7	100	18	0.20	No
	26	M	69	66	13	Cardiac	Yes	Yes	Yes	5	4	5	11	140	48	6	1.1	25	6	0.51	Yes
	27	F	37	52	13	Cardiac	Yes	Yes	Yes	6	5	9	27	57	234	6	1.4	100	7	0.58	No
	28	M	75	44	10	Visceral	Yes	Yes	Yes	22	20	24	53	203	42	5	1.4	86	24	0.29	Yes

Total (n = 21)		17 M(81%)	73 (56–78)	57 (50–65)	13 (10–15)	12 cardiac (57%)	17 (81%)	20 (95%)	21 (100%)	5 (4–9)	4 (3–10)	7 (5–12)	26 (14–39)	140 (86–204)	48 (41–69)	6 (5–6)	1.5 (1.3–1.8)	100 (73–100)	12 (7.5–24)	0.20 (0.10–0.32)	12 (57%)

Patients were classified into two groups (PF4^+ ^and PF4^-^) according to anti-PF4/heparin antibody concentration (optical density [OD] > 1 IU or not). PF4^+ ^patients 1 to 4 also had a positive functional test for heparin-induced thrombocytopenia (platelet-rich plasma aggregation test). No differences between these four patients and the remaining three patients with a negative functional test were observed. The types of surgery for the two trauma patients were orthopedic and visceral for patient 5 and visceral surgery only for patient 15. Shock was defined as the need for vasopressor support. Time to onset is the time between intensive care unit (ICU) admission or continuous veno-venous hemofiltration (CVVH) or unfractionated heparin (UFH) and the day of anti-PF4/heparin antibody assay. The Four T-score was calculated by considering that hemofiltration-filter clotting did constitute a 'recurrent thrombosis' (= 1 point) [9]. Results are expressed as median (first quartile to third quartile) or number. ^a^*P *< 0.05. M, male; F, female; aPTT, activated partial thromboplastin time (observed during the continuous veno-venous hemofiltration session with hemofiltration-filter clotting); D_0_, day of admission; D_PF4_, day of anti-PF4/heparin antibody assay; MV, mechanical ventilation; PF4^-^, anti-PF4/heparin antibody-negative; PF4^+^, anti-PF4/heparin antibody-positive; SAPS II, Simplified Acute Physiologic Score [19]; SOFA, Sepsis-related Organ Failure Assessment [20].

### Platelet counts

The platelet counts and platelet count variations did not differ between the two groups at any time point (Tables 1 and 2). In four patients (one PF4^+ ^and three PF4^-^), the platelet count was higher than 100 × 10^9^/L the day of the anti-PF4/heparin antibody test (Table 1).

**Table 2:** Laboratory and continuous veno-venous hemofiltration (CVVH) parameters

	PF4^+ ^(n = 7)	PF4^- ^(n = 21)	*P *value
Creatinine before first CVVH, μmol/L	600 (247–742)	403 (312–441)	0.34
Urea before first CVVH, mmol/L	17 (10–25)	15 (12–24)	0.91
Number of CVVH sessions	5 (4–11)	5 (3–9)	0.65
Mean CVVH duration^a^, hours	10.4 (9.6–18–4)	22.7 (15.7–22.3)	0.03
Mean urea reduction ratios^a^, percentage	17 (10–37)	44 (30–52)	0.04
Platelet nadir, 10^9^/L	40 (24–46)	35 (17–43)	0.54
Maximum platelet count, 10^9^/L	324 (238–366)	286 (182–403)	0.81
PF4 platelet count variation (D_0_-D_PF4_), percentage	59 (35–73)	56 (35–73)	0.98
Maximum platelet count variation (D_0_-D_nadir_), percentage	77 (49–81)	79 (55–87)	0.81
Mean minimum aPTT ratios^b^	1.20 (1.12–1.22)	1.35 (1.10–1.56)	0.35
Mean maximum aPTT ratios^b^	1.85 (1.65–2.12)	2.19 (1.51–2.93)	0.35

Platelet count variations were calculated as follows: [(platelet D_0 _– platelet D_x_)/platelet D_0_] × 100, where platelet D_0 _is the platelet count on admission and platelet D_x _is the platelet count on the day of anti-PF4/heparin antibody assay (D_PF4_) or the minimal platelet count (D_nadir_). Results are expressed as median (first quartile to third quartile). ^a^For PF4^+ ^patients, mean duration and efficiency of CVVH sessions before replacement of unfractionated heparin by danaparoid; ^b^means of minimum and maximum aPTT ratios observed during all the CVVH sessions before D_PF4_. aPTT, activated partial thromboplastin time (on heparin therapy during all continuous veno-venous hemofiltration sessions); PF4^-^, anti-PF4/heparin antibody-negative; PF4^+^, anti-PF4/heparin antibody-positive.

### Continuous veno-venous hemofiltration sessions

**Figure 1:**
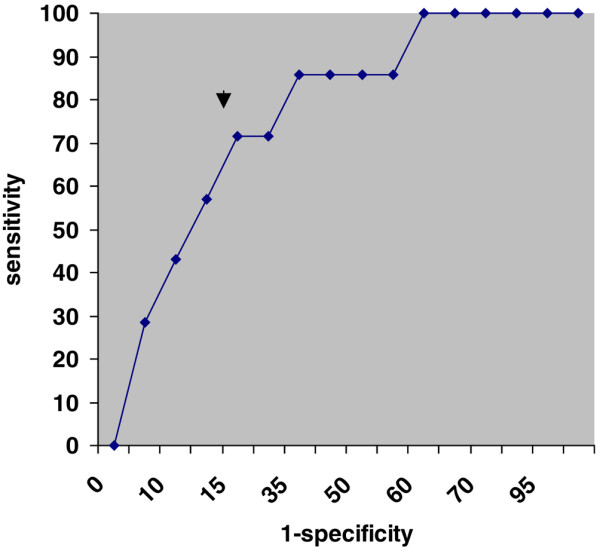
Receiver operator characteristic curve for continuous veno-venous hemofiltration (CVVH) duration. A receiver operating characteristic curve was used to assess the best cutoff for time to hemofiltration-filter clotting to predict a positive PF4 test. The arrow shows that a 6-hour duration of CVVH session is the most accurate cutoff (sensitivity 71%, specificity 85%, and area under the curve 0.83).

**Figure 2:**
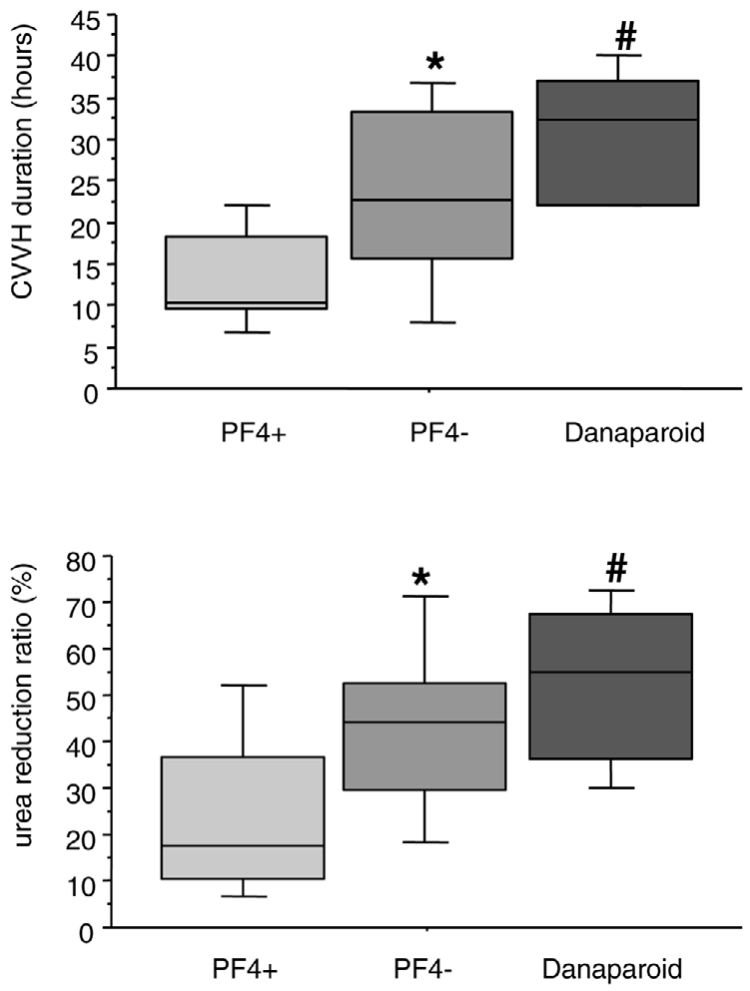
Duration and efficiency of continuous veno-venous hemofiltration (CVVH) sessions. Boxes represent medians, interquartile ranges, and 10th and 90th percentiles of mean duration of CVVH sessions (upper panel) and urea reduction ratios ([(urea before – urea after)/urea before] × 100, lower panel) observed in PF4^+ ^(n = 7) and PF4^- ^(n = 21) patients when using unfractionated heparin (50 and 132 sessions for PF4^+ ^and PF4^- ^patients, respectively) and in PF4^+ ^patients when using danaparoid sodium for anticoagulation (17 sessions). *P < 0.05 compared with PF4^+ ^(using a Mann-Whitney test); ^#^*P *= 0.027 compared with PF4^+ ^(using a Wilcoxon rank test). There was no difference between PF4^- ^and danaparoid CVVH durations (*P *= 0.17) or urea reduction (*P *= 0.27). PF4^-^, anti-PF4/heparin antibody-negative; PF4^+^, anti-PF4/heparin antibody-positive.

The main CVVH session characteristics are summarized in Tables 1 and 2. No significant difference was observed in terms of aPTT ratios, regardless of the value considered. The duration of CVVH sessions was significantly decreased in PF4^+ ^patients (Table 1; *P *= 0.007). An inverse correlation was observed between the anti-PF4/heparin antibody concentration (OD) and the duration of CVVH sessions (*r*^2 ^= 0.24; *P *= 0.01). As CVVH session duration was the only parameter associated with positive antibodies, we assessed the most relevant duration to predict a positive test. The ROC curve analysis showed that 6 hours was the best cutoff to predict a positive anti-PF4/heparin antibody test (with a sensitivity of 71%, a specificity of 85%, and an area under the curve of 0.83) (Figure 1). The efficiency of CVVH sessions (assessed by the urea reduction ratio) was also decreased in PF4^+ ^patients (Figure 2). The use of danaparoid sodium as a replacement for UFH allowed the restoration of adequate CVVH duration (32 [22 to 37] hours; *P *< 0.05) and efficiency (urea reduction 55% [36% to 68%]; *P *< 0.03) for PF4^+ ^patients (Figure 2).

## Discussion

Repeated hemofiltration-filter clotting leading to anti-PF4/heparin antibody assay is frequent in our experience. We report a large proportion of patients with positive anti-PF4/heparin antibodies (7 out of 28). A CVVH session lasting less than 6 hours was associated with positive antibodies. As the aim of this study was not to assess the prevalence of HIT or positive anti-PF4/heparin antibodies in our overall ICU population, we did not measure anti-PF4/heparin antibody for patients without hemofiltration-filter clotting. However, it is unlikely that 'unselected' patients would have a high rate of positive anti-PF4/heparin antibodies. Indeed, the incidence of HIT in intensive care patients is about 1% [6,10]. Furthermore, to our knowledge there are no data in the literature to support routine anti-PF4/heparin antibody assays without a clinical suspicion of HIT or thrombosis/clotting on heparin therapy [2,6].

We and others have already described cases of repeated hemofiltration-filter clotting associated with positive anti-PF4/heparin antibodies [7,8]. However, such a large proportion of patients with positive anti-PF4/heparin antibodies was not expected and has not been previously reported. In their large cohort of 2,046 post-operative critically ill patients, Gettings and colleagues [10] reported only 19 (0.9%) patients with positive anti-PF4/heparin antibodies. The presence of positive antibodies may be an independent risk factor for thrombotic events, apart from the diagnosis of HIT [11], as anti-PF4/heparin antibodies are associated with dose-dependent activation of coagulation [12]. We therefore decided to use a high cutoff value for positive antibody OD (> 1 IU). It should be emphasized that the anti-PF4/heparin antibody OD observed in our patients was inversely correlated with the CVVH session duration. The time to onset of hemofiltration-filter clotting appeared to be shorter in patients with higher anti-PF4/heparin antibody titer, possibly indicating more intense activation of coagulation.

Despite the high cutoff chosen to define positive antibodies, a large number of patients presented positive antibodies. This is probably related to our study population as the probability of HIT (or positive anti-PF4/heparin antibodies) depends on the population studied [2,9,13]. In our study, patients were highly selected and represented less than one third of all patients undergoing CVVH in our unit. Furthermore, complicated cardiac surgery patients account for the majority of our case mix and, together with orthopedic patients, are known to present the highest risk of positive anti-PF4/heparin antibodies [2,14,15].

We do not assume that all seven anti-PF4/heparin antibody-positive patients had true HIT, but the diagnosis of HIT was very likely in four of these seven patients and was probable in the remaining three patients according to published criteria [6]. Furthermore, a strong positive test result (OD > 1) is associated with a high likelihood ratio for HIT [2]. The diagnosis of HIT is based on two aspects: clinical features, including the course of platelet count (with a greater than 50% decrease over a typical time scale [16]), and laboratory features, including confirmation tests [2,17]. In our study population, the timing of HIT suspicion was within the usual range. Moreover, the Four T-scores [9], though not validated for ICU patients, indicated at least an intermediate risk for all patients. Although thrombocytopenia is the most common feature of HIT, it is also a very common finding in ICU patients and could be related to many causes, including CVVH itself. In fact, platelet counts were low in both groups (PF4^+ ^and PF4^-^). The time to clotting of the hemofiltration-filter therefore appeared to be the only factor associated with positive anti-PF4/heparin antibodies. Interestingly, the 6-hour cutoff reported here is consistent with the 7.5-hour interval before clotting observed by Samuelsson and colleagues [7].

Finally, although the diagnosis of HIT was not certain for all anti-PF4/heparin antibody-positive patients, replacement of UFH by danaparoid sodium allowed the restoration of CVVH duration and efficiency, supporting the clinical relevance of anti-PF4/heparin antibody assay in the case of repeated hemofiltration-filter clotting, at least in a surgical ICU patient population. Interestingly, the durations of CVVH sessions under danaparoid sodium we observed were close to those described by de Pont and colleagues [18]. In accordance with the latter authors, we did not observe any bleeding complications during danaparoid therapy. Furthermore, once the diagnosis of HIT is considered, all heparin exposure, including intravenous catheter locks, should cease.

Because of the retrospective nature of the study, we are not able to precisely determine the causes of hemofiltration-filter clotting in the remaining 21 patients with negative anti-PF4/heparin antibody. In particular, we did not perform PRP aggregation assays or serotonin release assays to search for HIT with an antigenic target different from PF4, nor did we assess the anti-thrombin III levels of these patients, but they had elevated aPTT ratios, indicating a certain activity of UFH. Further studies focusing on hemofiltration-filter clotting are needed to better describe this issue and the different factors associated with filter clotting (such as coagulation activation or abnormalities as well as CVVH settings or catheter dysfunction).

## Conclusion

We report a large proportion of anti-PF4/heparin antibody-positive patients in the case of repeated hemofiltration-filter clotting during CVVH, particularly when clotting occurred in less than 6 hours. In these cases, replacement of UFH by danaparoid sodium (or other agents such as citrate or saline flushes) may be useful.

## Key messages

• Heparin-induced thrombocytopenia diagnosis and positive anti-PF4/heparin antibody were frequently observed in the case of repeated hemofiltration-filter clotting during continuous veno-venous hemofiltration (CVVH) under heparin.

• Hemofiltration-filter clotting in less than 6 hours was the best predictor of positive anti-PF4/heparin antibody, while platelet count and its variation were not associated with a positive test.

• The replacement of heparin by danaparoid sodium allowed the restoration of CVVH duration and efficiency in anti-PF4/heparin antibody-positive patients.

## Abbreviations

aPTT = activated partial thromboplastin time; CVVH = continuous veno-venous hemofiltration; HIT = heparin-induced thrombocytopenia; ICU = intensive care unit; OD = optical density; PF4^- ^= anti-PF4/heparin antibody-negative; PF4^+ ^= anti-PF4/heparin antibody-positive; PRP = platelet-rich plasma; ROC = receiver operating characteristic; UFH = unfractionated heparin.

## Competing interests

The authors declare that they have no competing interests.

## Authors' contributions

SL helped to design the study, to treat the patients, to review the charts of the patients, was responsible for the statistical analysis and drafted the manuscript. PP helped to design the study, to treat the patients, to review the charts of the patients and drafted the manuscript. NA was responsible for the laboratory assays and drafted parts of the manuscript AG helped to treat the patients, to review the charts of the patients and to review the manuscript. AB helped to treat the patients, to review the charts of the patients and to review the manuscript. PM helped to treat the patients and drafted the manuscript.
